# The SHH/Gli axis regulates CD90‐mediated liver cancer stem cell function by activating the IL6/JAK2 pathway

**DOI:** 10.1111/jcmm.13651

**Published:** 2018-05-02

**Authors:** Ketao Zhang, Siyao Che, Chuzhi Pan, Zheng Su, Shangyou Zheng, Shanglin Yang, Huayao Zhang, Wenda Li, Weidong Wang, Jianping Liu

**Affiliations:** ^1^ Guangdong Provincial Key Laboratory of Malignant Tumor Epigenetics and Gene Regulation Sun Yat‐Sen Memorial Hospital Sun Yat‐Sen University Guangzhou China; ^2^ Department of Hepatopancreatobiliary Surgery Sun Yat‐sen Memorial Hospital of Sun Yat‐sen University Guangzhou China; ^3^ Department of Hepatobiliary Surgery Gaozhou People's Hospital Gaozhou China; ^4^ Department of Hepatobiliary Surgery the Third Affiliated Hospital of Sun Yat‐sen University Guangzhou China; ^5^ Department of Hepatobiliary Surgery Shunde Hospital of Southern Medical University Foshan Guangdong Province China

**Keywords:** CD90, Gli13, Janus kinase 2, liver cancer, signal transducer and activator of transcription 3, Sonic Hegdehog, stem cell

## Abstract

The cell surface antigen CD90 has recently been established as a promising marker for liver cancer stem cells. This study aimed to investigate potential implications of SHH/Gli signalling in CD90+ liver cancer stem cells. Correlation of the expression of SHH signalling components and CD90 in liver cancer cells and clinical tissues, as well as in enriched CD90+ liver cancer stem cells and the TCGA database, were analysed by quantitative RT‐PCR, Western blotting and flow cytometry. Functional analysis was conducted by siRNA‐mediated CD90, Gli1 and Gli3 gene knockdown, SHH treatment and application of the JAK2 inhibitor AZD1480 and IL6 neutralizing antibody in CD90+ liver cancer stem cells, followed by cell proliferation, migration, sphere formation and tumorigenicity assays. CD90 expression exhibited a high positive correlation with Gli1 and Gli3 in multiple liver cancer cell lines and human cancerous liver tissues, both of which showed a significant increase in liver cancer. Analysis of TCGA data revealed an association of CD90, Gli1 and Gli3 with a short overall survival and positive correlation between CD90 expression and Gli3 expression level. The stem cell potentials of CD90+ 97L liver cancer cells were greatly impaired by Gli1/3 knockdown with siRNA but enhanced by SHH treatment. Application of the JAK2 inhibitor AZD1480 and IL6 neutralizing antibody showed the CD90 and SHH/Gli‐regulated liver cancer stem cell functions were mediated by the IL6/JAK2/STAT3 pathway. The stem cell properties of CD90+ liver cancer cells are regulated by the downstream SHH/Gli and IL6/JAK2/STAT3 signalling pathways.

## 1 INTRODUCTION

Liver cancer is one of the severe human malignant diseases and is listed as the second most common cause of tumor‐related death. More seriously, the incidence and mortality of liver cancer have been increasing in recent years, with an estimated annual death rate of approximately 700 000 patients worldwide in 2017, especially in East Asian countries.^1^, ^2^ Hepatocellular carcinoma (HCC) is the most common subtype of liver cancer, comprising over 90% of human liver cancers, and it can be etiologically attributed to multiple factors such as chronic infection with hepatitis B virus or hepatitis C virus, alcoholic liver diseases and metabolic deregulation conditions such as hemochromatosis.^3^ Curative therapies such as liver transplantation and resection are only effective for early‐stage tumours, but not for patients with more advanced stages.^1^ Progress in liver cancer biology has revealed the involvement of various somatic genetic alterations responsible for cell transformation and liver cancer progression, and the great complexity of molecular mechanisms underlying its subtyping, diagnosis, resistance and recurrence, underscoring the pressing need for further investigation.

The discovery of cancer stem cells (CSCs) over the last decade has created great interest among the research community because of their potential associations with cancer initiation and progression, and also potentially with chemotherapy resistance, prognosis and recurrence.^4^ Stem cells in malignant tissues might provide a new impetus for cancer biology basic research, effective diagnosis and treatment strategies. Liver cancer stem cells, also termed hepatic CSCs, were detected as a cell population that was stained with the DNA‐binding dye Hoechst 33342, thus demonstrating representative cancer cell properties.^5^ Subsequently, numerous cell surface proteins, such as CD90, CD133 and CD44, were established as reliable biomarkers for liver cancer stem cells.^6^ CD90, also known as Thy‐1, is a glycosylphosphatidylinositol (GPI)‐anchored glycoprotein that is expressed in a number of cell types such as leucocytes, neurons, endothelial cells, fibroblasts and T cells.^7^ Functional investigations have shown that CD90 is a critical regulator of pleiotropic cellular events, including cell migration and death, cell–cell interactions, tumour growth, modulation of T cell activity and neurite outgrowth, thus demonstrating a close association with multiple physiological and pathological processes, including tumorigenesis, metastasis, nerve regeneration, fibrosis and inflammation.^7^

Previously, it has been shown that CD90 is expressed in hepatic stem/progenitor cells (HSPCs) and bone marrow mesenchymal stem cells,^8^, ^9^ including primarily cultured CD133+ glioblastoma stem cells,^10^ murine breast cancer stem cells 11 and hepatic cancer stem cells.^6^ The CD90+ stem cells from all liver cancer tissue specimens display a strong tumorigenic capacity after being transplanted into immunodeficient mice and thus have been established as a new potential marker for liver cancer disease surveillance.^6^ By comparing the gene expressional profiles between normal and tumor CD90+ cells, the expression of more than 500 genes was found to be regulated by CD90 in hepatic progenitors, including a large group of genes that are implicated in inflammation, drug resistance and lipid metabolism.^12^ However, the molecular pathways responsible for the CD90‐mediated tumorigenic capacity of liver cancer cells are still poorly understood.

The Sonic Hegdehog/glioma‐associated oncogene 1 (SHH/GL1) pathway is involved in pattern formation regulation during early embryonic development, stem cell proliferation in the late developmental phase and in cancer stem cells.^13^ The SHH/GLI‐related regulatory mechanisms have been shown to be important for the maintenance of cell homeostasis during postnatal development and adult tissue growth, as well as tumorigenesis, at least partially through cancer stem cell regulation mediated by autophagy‐related processes.^13^ Previous reports have demonstrated that the SHH/Gli pathway is involved in liver cancer development and is responsible for HCC cell growth and dedifferentiation.^14^, ^15^ However, the relationship between the SHH/Gli pathway and the regulation of CD90+ liver cancer stem cell functions, as well as the underlying molecular mechanisms, have not yet been addressed.

To gain more insights into the tumorigenic capacity of CD90+ stem cells in liver cancer, the correlation of major SHH/Gli signalling components such as Gli1 and Gli3 with CD90 expression in multiple liver cancer cell lines and tissue samples was evaluated in this study. Additionally, the functions of glioma‐associated oncogene 1 (GLI1) and GLI3 in liver cancer were further evaluated by depletion or activation of their expression in CD90+ cancer stem cells, and also the downstream signalling involving the interleukin‐6/Janus kinase 2 (IL6/JAK2) pathway and signal transducer and activator of transcription 3 (STAT3) phosphorylation, which might promote the application of CD90+ stem cells for liver cancer diagnosis and treatment.

## 2 MATERIALS AND METHODS

### 2.1 Cell lines and tissue samples

>The liver cancer cell lines LO2, hepG2, Huh7, SK‐Hep1, LM3 and MHCC‐97L were obtained from Guangzhou Cellcook Biotech CO., Ltd. These cancerous cells were cultured with DMEM supplemented with 0.1% foetal bovine serum (FBS) at 37°C in a culture chamber supplied with 5% CO_2_. Sonic Hedgehog (SHH) used for cell treatment (0.4 g/mL; 48 hours) in this study was purchased from Sigma‐Aldrich (SRP3156). Liver cancer tissue samples and paired adjacent normal tissues were collected from liver cancer patients who were hospitalized at the Department of Hepatopancreatobiliary Surgery, Sun Yat‐sen Memorial Hospital of Sun Yat‐sen University. Tissue collection and subsequent operations were confirmed by the Ethics Committee of Sun Yat‐sen University. Written informed consent was signed by all patients. The clinical samples were confirmed by experienced clinical pathologists prior to further analysis.

### 2.2 Quantitative RT‐PCR

Relative mRNA levels in liver cancer cells or tissue samples were determined by quantitative real‐time polymerase chain reaction (qRT‐PCR). Prior to RNA extraction, liver cancer cells were washed 3 times with PBS buffer and collected by centrifugation. Liver cancer tissues were homogenized in liquid nitrogen for RNA extraction. Total RNAs from cell lines and liver tissues were extracted using the PureLink™ RNA Mini Kit (Cat#12183018A; Thermo Fisher Scientific, USA) according to the manufacturer's instructions. Approximately 2 μg RNA was used for cDNA synthesis using the High‐Capacity cDNA Reverse Transcription Kit (Cat#4368813; Thermo Fisher Scientific) according to the protocol provided by the manufacturer. The relative expression levels were then determined by RT‐PCR using the SYBR Select Master Mix kit (Cat#4472908; Applied Biosystems) following the manufacturer's instructions. For relative quantitation, GAPDH was used as the internal control, and 3 independent replicates were carried out for statistical analysis.

### 2.3 Flow cytometry

To analyze the percentages of CD90+ cells in liver cancer cells, flow cytometry was performed following cell labelling with CD90‐FITC, human (clone: REA897) according to the manufacturer's instructions (order no. 130‐114‐901; Miltenyi Biotec). Cells were first washed 3 times with PBS buffer and then labelled with CD90 antibody. The cell subpopulation percentages were determined by flow cytometry using the MACSQuant^®^ Analyzer as described by the manufacturer. For quantitative analysis of cell percentages, at least 3 biological replicates were assessed.

### 2.4 Magnetic‐activated cell sorting (MACS)

The CD90+ liver cancer stem cells were separated using CD90 MicroBeads (order no. 130‐096‐253; Miltenyi Biotec) according to the manufacturer's instructions.^16^ Briefly, cultured liver cancer cells were washed with PBS buffer, digested with trypsin and labelled with CD90 MicroBeads at 4°C for 40 minutes in PBS buffer supplied with 2% bovine serum albumin and 10 mmol/L ethylenediaminetetraacetic acid. After 3 washes, cell sorting was performed using a MiniMACS column. CD90+ and CD90− liver cancer cells were separately collected and counted using a cell counter under microscopy.

### 2.5 Western blotting

The relative protein levels were analysed in this study by Western blotting using specific primary antibodies. Liver cancer cells were washed 3 times with PBS buffer and then subjected to total protein extraction. After boiling with protein loading buffer at 100°C for 5 minutes, approximately 25 g protein from each sample was separated by SDS‐PAGE, transferred onto PVDF membrane, blocked with 5% lipid‐free milk for 2 hours at room temperature, incubated with TBST‐containing primary antibodies for 1.5 hours, washed 3 times with TBST for 10 minutes, incubated with TBST containing secondary antibodies for 1 hours, washed 3 times again with TBST and finally developed with ECL solution (Amersham™). The primary antibodies used in this study against GLI1 (#ab49314), GLI3 (#ab126852), GAPDH (#ab8245), IL6 (#ab6672), phosphorylated JAK2 (#ab32101), phosphorylated STAT3 (#EP2147Y) and SOX2 (#ab97959) were purchased from the Abcam company. At least 3 independent biological repeats were performed for quantitation of protein abundances, and GAPDH was applied as the internal standard.

### 2.6 Immunohistochemistry

The protein levels of CD90, Gli1 and Gli3 in liver cancer tissues from 51 liver cancer patients were determined by immunohistochemistry using a standard immunohistochemistry protocol for paraffin‐embedded tissue sections. Briefly, the liver cancer tissue and adjacent normal tissue sections were deparaffinized with xylene and rehydrated in a gradient of alcohol solutions. The tissue sections were then incubated with 3% H2O2 for 15 minutes, incubated with blocking buffer for 1 hours, and then incubated with anti‐CD90, Gli1 or Gli3 antibody solutions for 1 hours. After 3 washes with PBS buffer, the liver tissue sections were then incubated with secondary antibody (biotinylated), streptavidin and diaminobenzidine reagent (Vector Laboratories, Canada).

### 2.7 TCGA data analysis

The correlation of CD90, Gli1 and Gli3 expression levels with the overall survival of liver cancer patients was evaluated using The Human Protein Atlas website (http://www.proteinatlas.org) based on expression data collected from The Cancer Genome Atlas (TCGA), as described previously.^17^ Transcriptomics data from, 365 liver cancer patients, 119 women and 246 men, 170 patients in stage I, 84 patients in stage II, 83 patients in stage III and 4 patients in stage IV, were used to analyse the correlation with overall survival. The higher survival probability of patients with low compared with high expression levels of target protein indicates that target gene expression level is negatively correlated with the overall survival of liver cancer patients. The correlation between CD90 expression level and Gli1 or Gli3 expression level was judged by comparison with the median FPKM (number of Fragments Per Kilobase of exon per Million reads). A *P*‐value < .05 indicated a positive correlation.

### 2.8 siRNA transfection

For knockdown of Gli1 and Gli3 expression in liver cancer cells, 97L cells with CD90 expression were transfected with siRNAs targeting Gli1 and Gli3 genes as previously described.^18^ Briefly, 97L liver cancer cells enriched using the MACS method were cultured in DMEM with FBS in 6‐well plates and then transfected with Gli1 or Gli3 siRNAs for 24 hours using the Lipofectamine RNAi MAX transfection kit according to the manufacturer's instructions (Invitrogen, USA). Liver cancer cells were harvested 48 hours after successful transfection, and the Gli1 and Gli3 protein levels were measured by Western blotting. The cells treated with Lipofectamine 2000 without siRNA were used as the negative control (NC) in this study. At least 3 biological replicates were performed for statistical analysis.

### 2.9 MTS proliferation, sphere formation, migration and tumorigenicity assays

The proliferation of liver cancer cells was evaluated using the MTS Cell Proliferation Assay Kit (Colorimetric) (No. 197010; Abcam, UK) following the manufacturer's instructions. Briefly, liver cancer cells seeded in 96‐well microtitre plates were cultured for approximately 48 hours and then mixed with MTS reagent (20 μL/well) and incubated for 2 hours at 37°C. Cell proliferation was finally determined by measuring the OD490 using a plate reader. At least 3 independent repeats were performed for statistical analysis. The sphere formation capacity,^18^ migration rate during the transwell assay 19 and in vivo tumorgenecity using non‐obese diabetic/severe combined immunodeficiency (NOD/SCID) mice 20 of liver cancer stem cells were analysed as previously described.

### 2.10 Statistical analysis

Statistical analysis in this study was performed using the SPSS software package (version 18.0, SPSS). Student's *t* test was carried out to evaluate the significance of differences among data from at least 3 biological repeats. A *P* value < .05 or <.01 was used to define a significant or extremely significant difference, respectively.

## 3 RESULTS

### 3.1 Correlated expression of CD90, Gli1 and Gli3 in liver cancer cells

**Figure 1:**
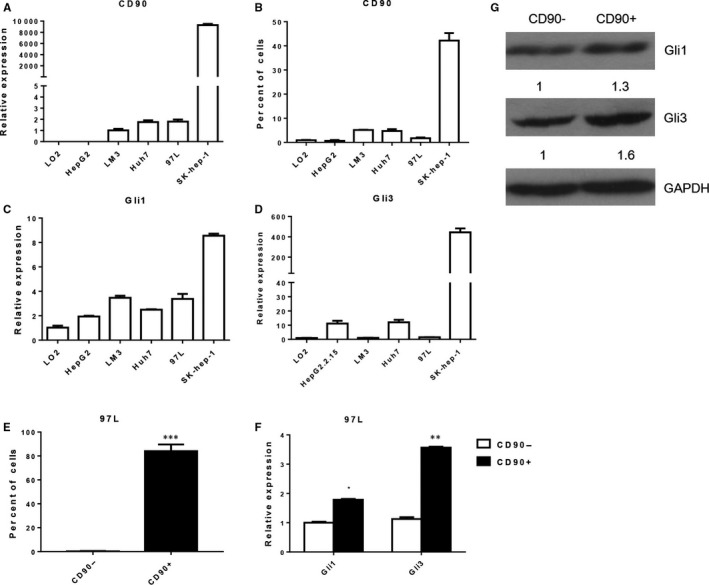
Correlated expression of CD90, Gli1 and Gli3 in liver cancer cells. A, CD90 mRNA levels among different liver cancer cell lines. Quantitative RT‐PCR was performed to determine the CD90 expression level. B, Percentages of CD90+ cells among different liver cancer cell lines by flow cytometry. C, D, Relative mRNA levels of Gli1 and Gli3 among different liver cancer cell lines by quantitative RT‐PCR. E, Enrichment of CD90+ 97L cells by magnetic‐activated cell sorting (MACS). F, Expression of Gli1 and Gli3 in CD90‐positive and CD90‐negative 97L cells by quantitative RT‐PCR. G, Gli1 and Gli3 protein abundances in CD90‐positive and CD90‐negative 97L cells by Western blotting. GAPDH was used as the internal standard. Gli1: Glioma‐associated oncogene 1; GAPDH: glyceraldehyde‐3‐phosphate dehydrogenase. * indicates significant differences

To evaluate the expression correlation of CD90 and SHH/Gli signalling in liver cancer, the expression of CD90 and major components of this pathway were first determined in different liver cancer cell lines (Figure 1 and Figure S1). Quantitative RT‐PCR showed the different CD90 expression levels among LO2, HepG2, LM3, Huh7, 97L and Sk‐hep‐1 cell lines, revealing the highest expression level of CD90 (Figure 1A). The variation of CD90 expression among these liver cancer cell lines was validated by percentages of CD90‐positive cells, as shown by flow cytometry (Figure 1B). More importantly, the expression of Gli1 and Gli3 showed similar expression patterns in these liver cancer cell lines (Figure 1C,D). For further validation, CD90+ cells were enriched by magnetic‐activated cell sorting (MACS) from a 97L liver cancer cell culture, and nearly 80% of the cells were found to be CD90‐positive (Figure 1E). Consistently, the expression of both Gli1 and Gli3 was significantly increased in CD90+ 97L cells compared with CD90‐ cells (Figure 1F). Western blotting also showed a similar increase in Gli1 and Gli3 protein abundances in CD90+ 97L cells (Figure 1G).

### 3.2 CD90, Gli1 and Gli3 expression correlation in liver cancer tissues

**Figure 2:**
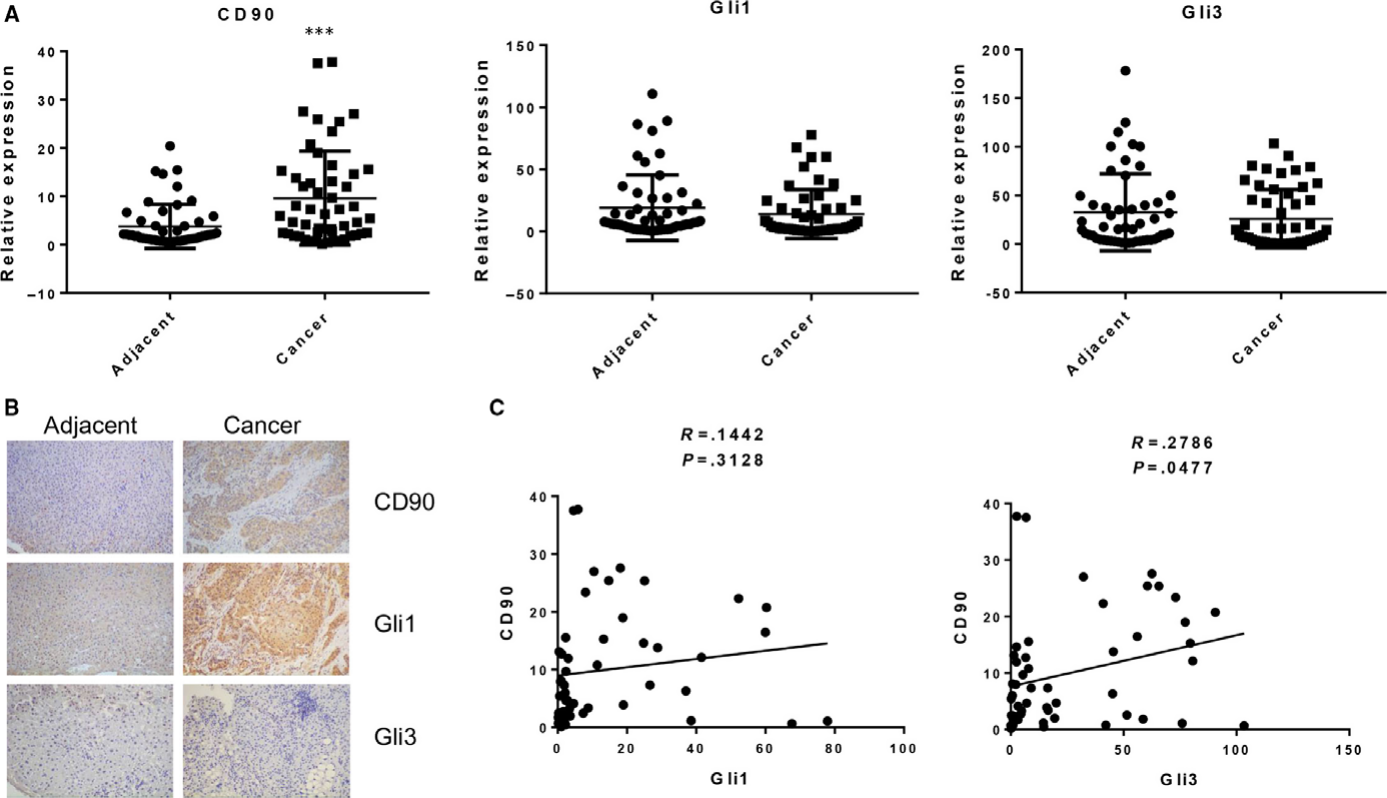
CD90, Gli1 and Gli3 expression in liver cancer tissues. A, CD90, Gli1, and Gli3 mRNA levels in liver tissues from 51 liver cancer patients by quantitative RT‐PCR. B, CD90, Gli1 and Gli3 proteins in liver cancer tissue by immunohistochemistry. C, The correlation of CD90 expression with Gli1 and Gli3 was validated in clinical liver cancer tissues… GAPDH was used as the internal standard. Gli1: Glioma‐associated oncogene 1; GAPDH: glyceraldehyde‐3‐phosphate dehydrogenase. * indicates significant differences

For further validation of the correlation expression of CD90, Gli1 and Gli3 in liver cancer cells, the expression levels of these 3 genes among 51 pairs of liver cancer tissues and corresponding adjacent normal tissues were analysed by quantitative RT‐PCR. We found that the CD90 mRNA level was elevated in the majority of clinical tumour tissues from liver cancer patients compared with the adjacent normal tissues (Figure 2A). However, no significant increase in Gli1 or Gli3 expression was observed in the whole collection of cancer tissues (Figure 2A), possibly because of the extensive individual complexity. In a case study using the immunohistochemistry (IHC) assay, we observed that the protein level of Gli1 was greatly elevated in cancer tissues with high CD90 expression (Figure 2B). We then re‐assessed the expression levels of Gli1 and Gli3 among these cancer tissues with high CD90 expression and observed elevated Gli1 and Gli3 expression in high‐CD90 liver cancer tissues (Figure 2C). The correlation of CD90 expression with Gli1 (*R* = .1442, *P* = .3128) and Gli3 (*R* = .2786, *P* = .0477) was also validated by these expression results in clinical liver cancer tissues.

### 3.3 Association of CD90, Gli1 and Gli3 with survival and malignancy in liver cancer patients

**Figure 3:**
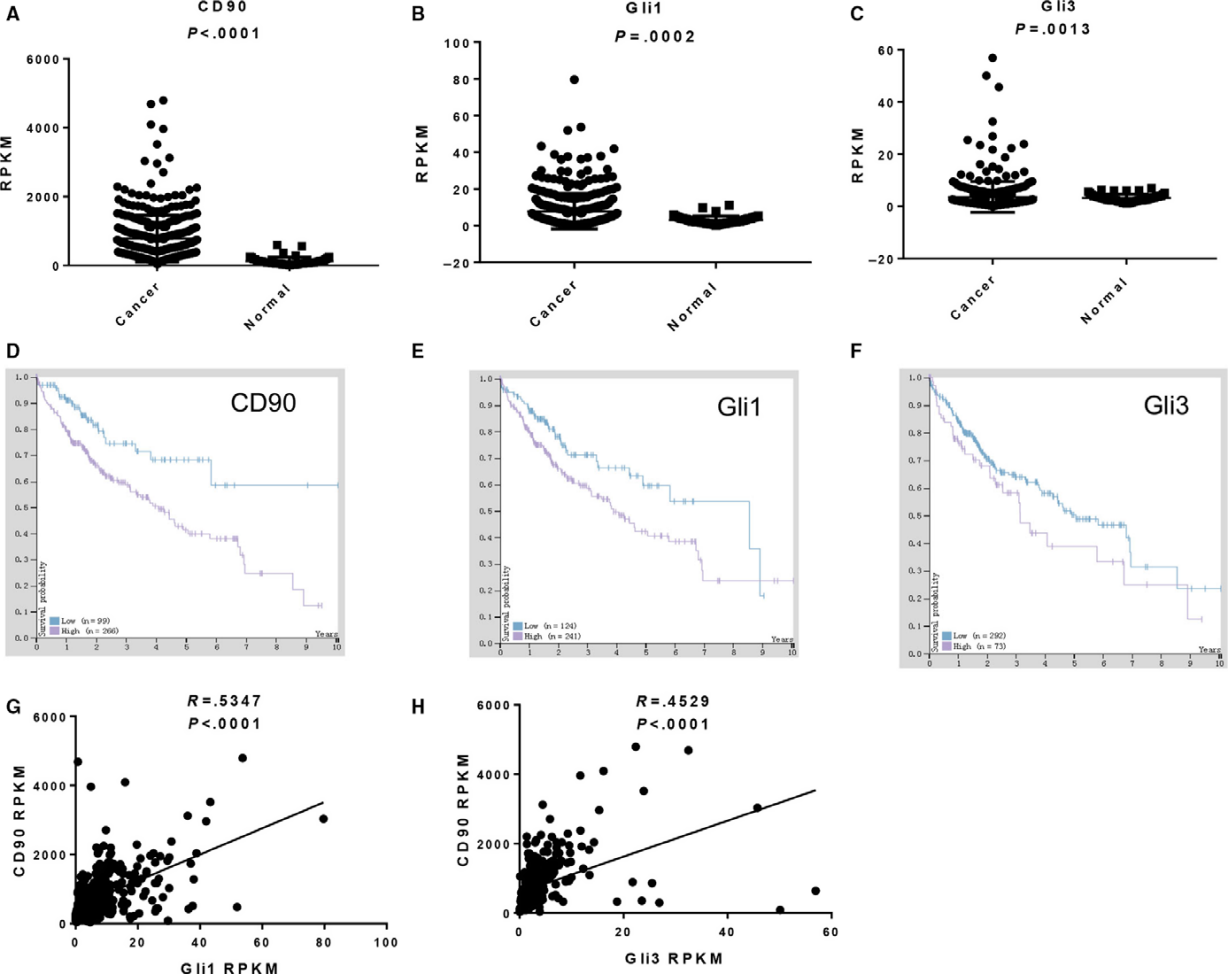
CD90, Gli1 and Gli3 expression in liver cancer tissues via the TCGA database. A‐C, Data from the Cancer Genome Atlas (TCGA) containing expression profiles of 365 liver cancer patients were analysed to measure the CD90 (A), Gli1 (B) and Gli3 (C) expression levels. D‐F, Correlation of CD90 (D), Gli1 (E) and Gli3 (F) expression with overall survival in liver cancer patients by Kaplan‐Meier survival plots. The *Y*‐axis shows the survival probability and the *X*‐axis shows the years of survival. Patient with high and low expression levels is shown in purple and blue, respectively. G & H. The correlation of CD90 expression with Gli1 (G) and Gli3 (H) was also validated in liver cancer tissues A significant difference was defined by *P* < .05. Gli1: Glioma‐associated oncogene 1; GAPDH: glyceraldehyde‐3‐phosphate dehydrogenase; TCGA: The Cancer Genome Atlas; FPKM: number Fragments Per Kilobase of exon per Million reads

For a more reliable correlation of CD90, Gli1 and Gli3 expression levels with liver cancer progression, pathological data from the Cancer Genome Atlas (TCGA) containing expression profiles of 365 liver cancer patients were further analysed as described in the Material and Methods section. We then assessed the expression levels of CD90, Gli1 and Gli3 among these cancer tissues and observed elevated CD90, Gli1 and Gli3 expression in liver cancer tissues (Figure 3A‐C). We also found that the average survival probability was higher for liver cancer patients with lower than with higher CD90 expression levels, demonstrating that high CD90 expression was correlated with a shortened overall survival in liver cancer patients (Figure 3D). A similar correlation was also observed for both Gli1 and Gli3 gene expression in liver cancer tissues (Figure 3E,F), showing that the expression of these 3 genes was associated with a poor prognosis of liver cancer patients. Additionally, the positive correlation of CD90 expression with Gli1 (*R* = .5347, *P* < .0001) and Gli3 (*R* = .4529, *P* < .0001) was also validated by these expression results in liver cancer tissues (Figure 3G,H). Consistent with the above‐described expression results, CD90, Gli1 and Gli3 expression levels were correlated in liver cancer tissues.

### 3.4 Gli1 and Gli3 depletion depresses stem cell properties of CD90+ liver cancer cells

**Figure 4:**
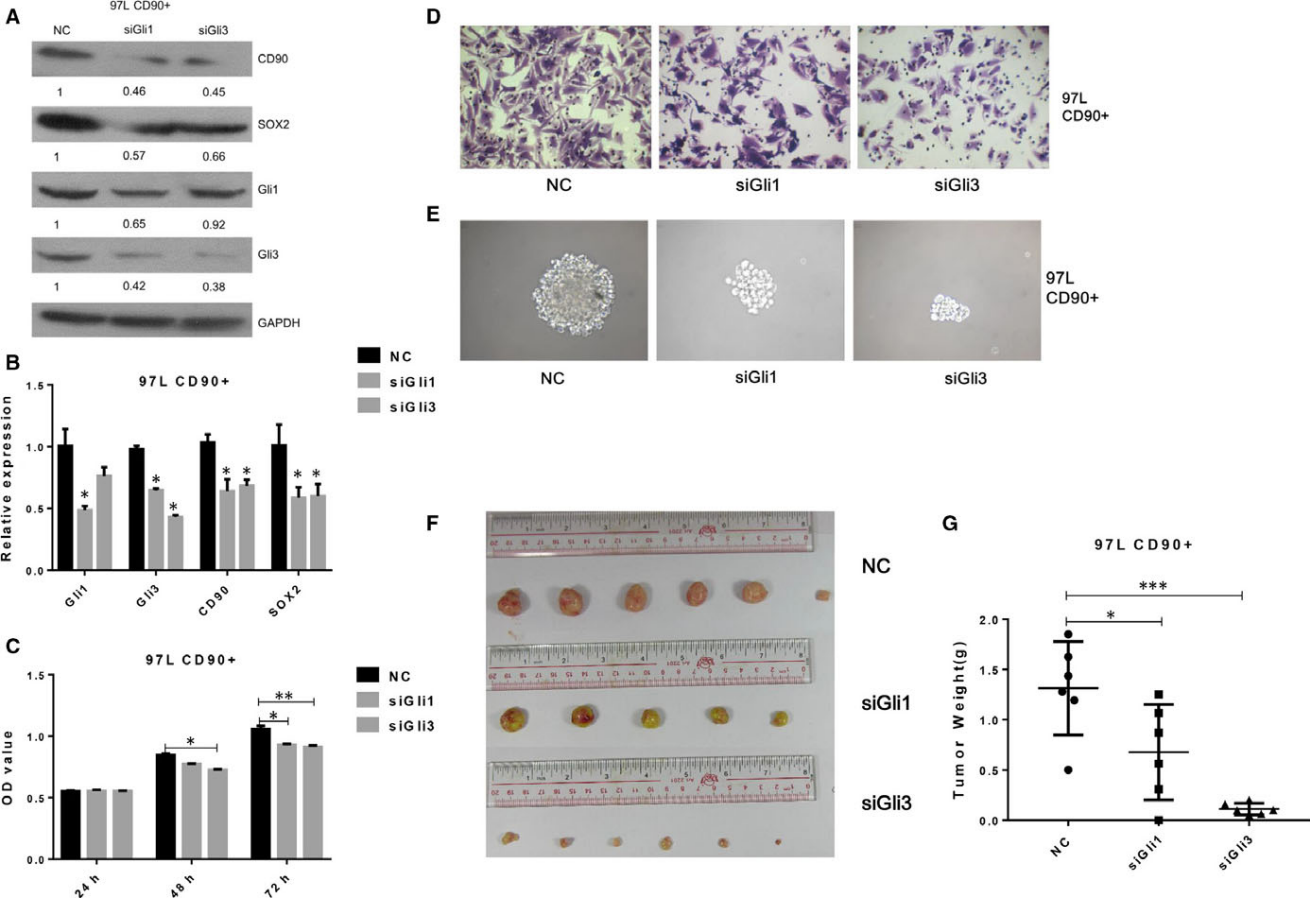
siRNA knockdown of Gli1 and Gli3 expression in CD90+ liver cancer cells. A & B, CD90, SOX2, Gli1 and Gli3 protein levels in CD90+ 97L liver cancer cells transfected with specific siRNAs. Western blotting and quantitative RT‐PCR were performed to measure the protein (A) and mRNA (B) abundance. GAPDH was applied as the internal standard. C, Proliferation rates of CD90+ liver cancer cells transfected with specific siRNAs by the MTS assay. D, Migration rates of CD90+ liver cancer cells transfected with specific siRNAs via the transwell assay. E, Sphere formation capacity of CD90+ liver cancer cells transfected with specific siRNAs. F & G, Tumorigenicity of CD90+ liver cancer cells transfected with specific siRNAs. Tumours were formed by inoculation of liver cancer cells into NOD/SCID mice. NC: negative control; siGli1: Gli siRNA; siGli3: Gli3 siRNA; SOX2: Sex Determining Region Y‐Box 2; NOD/SCID: non‐obese diabetic/severe combined immunodeficiency; GAPDH: glyceraldehyde‐3‐phosphate dehydrogenase. * indicates significant differences with *P* < .05

To analyse the role of Gli1 and Gli3 in the function of CD90+ liver cancer cells, knockdown of Gli1 and Gli3 expression was carried out using specific siRNAs in CD90+ 97L liver cancer cells. After siRNA transfection, the expression of Gli1 and Gli3 showed a significant decrease compared with the negative control (Figure 4A,B), demonstrating the efficient knockout of gene expression in liver cancer cells. The MTS assay showed that the proliferation rates of CD90+ liver cancer cells transfected with Gli1 or Gli3 siRNA were significantly decreased in comparison with the negative control (Figure 4C). Similarly, the transwell assay showed that the migration rates of CD90+ liver cancer cells were remarkably suppressed by transfection with Gli1 and Gli3 siRNAs (Figure 4D). Moreover, the sphere formation capacity of CD90+ liver cancer stem cells was also greatly inhibited by depletion of Gli1 and Gli3 expression As (Figure 4E). More importantly, the in vivo tumorigenicity assay showed that the sizes and weights of tumours originating from CD90+ liver cancer cells with suppressed Gli1 or Gli3 expression were markedly reduced compared with the negative control (Figure 4F,G). Our quantitative RT‐PCR and Western blotting experiments showed that the expression of CD90 was also significantly down‐regulated by knockdown of Gli1 and Gli3 expression in CD90+ liver cancer cells (Figure 4A,B). Furthermore, the expression of Sex Determining Region Y‐Box 2 (SOX2), the self‐renewal regulatory factors and major stem cell markers, was also greatly repressed by CD90 depletion in liver cancer cells (Figure 4A,B). These molecular and cellular assays fully validated that the stem cell properties of CD90+ liver cancer cells were mediated by Gli1 and Gli3 expression.

### 3.5 SHH treatment enhances liver cancer stem cell activity through CD90

**Figure 5:**
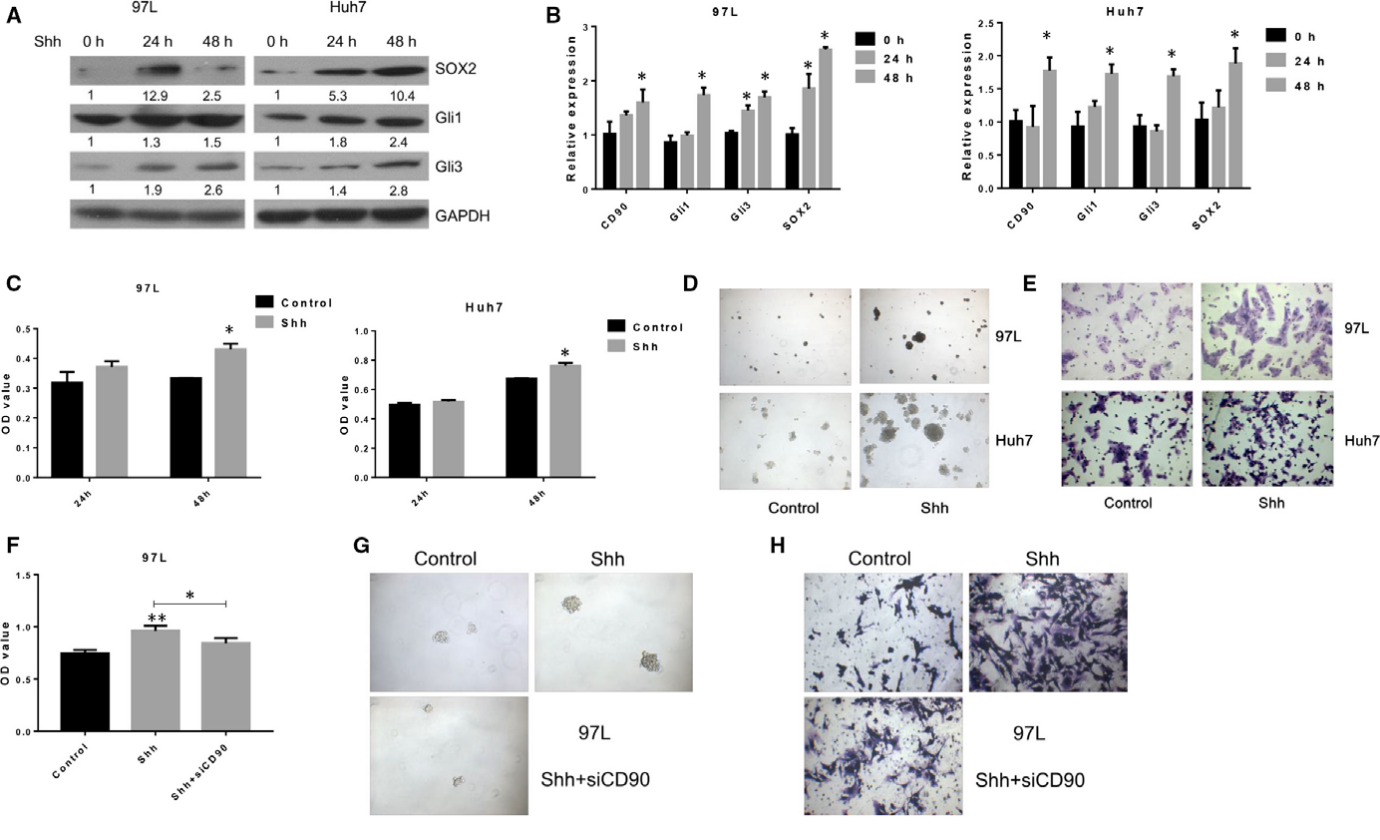
SHH treatment of CD90+ liver cancer cells. A & B, SOX2, Gli1 and Gli3 protein levels in CD90+ 97L and Huh7 liver cancer cells treated with SHH. Cells were treated with 0.4 g/mL SHH for 48 h, and Western blotting was performed to measure the protein abundance. GAPDH was used as the internal standard. C, Proliferation rates of CD90+ liver cancer cells in CD90+ liver cancer cells treated with SHH by the MTS assay. D, Sphere formation capacity of CD90+ liver cancer cells treated with SHH. E, Migration rates of CD90+ liver cancer cells in CD90+ liver cancer cells treated with SHH by the transwell assay. F, Proliferation rates of CD90+ liver cancer cells in CD90+ liver cancer cells treated with siCD90 and followed by SHH through the MTS assay. G, Sphere formation capacity of CD90+ liver cancer cells treated with siCD90 and followed by SHH. H, Migration rates of CD90+ liver cancer cells in CD90+ liver cancer cells treated with siCD90 and followed by SHH through the transwell assay. SHH: Sonic Hedgehog; GAPDH: glyceraldehyde‐3‐phosphate dehydrogenase; SOX2: Sex Determining Region Y‐Box 2. * indicates *P* < .05

For further exploration of the involvement of the SHH/Gli axis in liver cancer stem cell activity, CD90+ 97L and Huh7 liver cancer cells were treated with 0.4 g/mL SHH (SRP3156; Sigma‐Aldrich). As expected, protein and mRNA levels of Gli1 and Gli3 were greatly elevated in CD90+ liver cancer cells following SHH treatment Figure 5A,B). Consistent results were observed in CD90+ liver cancer cells treated with SHH, which were completely reversed compared with those transfected with Gli1 or Gli3 siRNA (Figures 4 and 5). Specifically, the proliferation rate was markedly higher in liver cancer cells treated with SHH than the control (Figure 5C); the sphere formation (Figure 5D) and migration capacity (Figure 5E) of CD90+ liver cancer cells were significantly enhanced by SHH treatment; and the expression of CD90 and SOX2 genes was remarkably increased by SHH treatment in liver cancer cells expressing CD90 (Figure 5A,B and Figure S2). After siCD90 transfection with 97L and then following SHH treatment, the proliferation rates (Figure 5F), sphere formation capacity (Figure 5G) and migration rates (Figure 5H) were inhibited by siCD90 treatment.

In combination with the suppressed stem cell properties of CD90+ liver cancer cells transfected with Gli siRNAs (Figure 4), these results persuasively demonstrated that the stem cell properties of CD90+ liver cancer cells were mediated by activation of SHH/Gli‐related signalling pathways during liver cancer progression.

### 3.6 IL6/JAK2 signalling and STAT3 phosphorylation in CD90+ liver cancer cells

**Figure 6:**
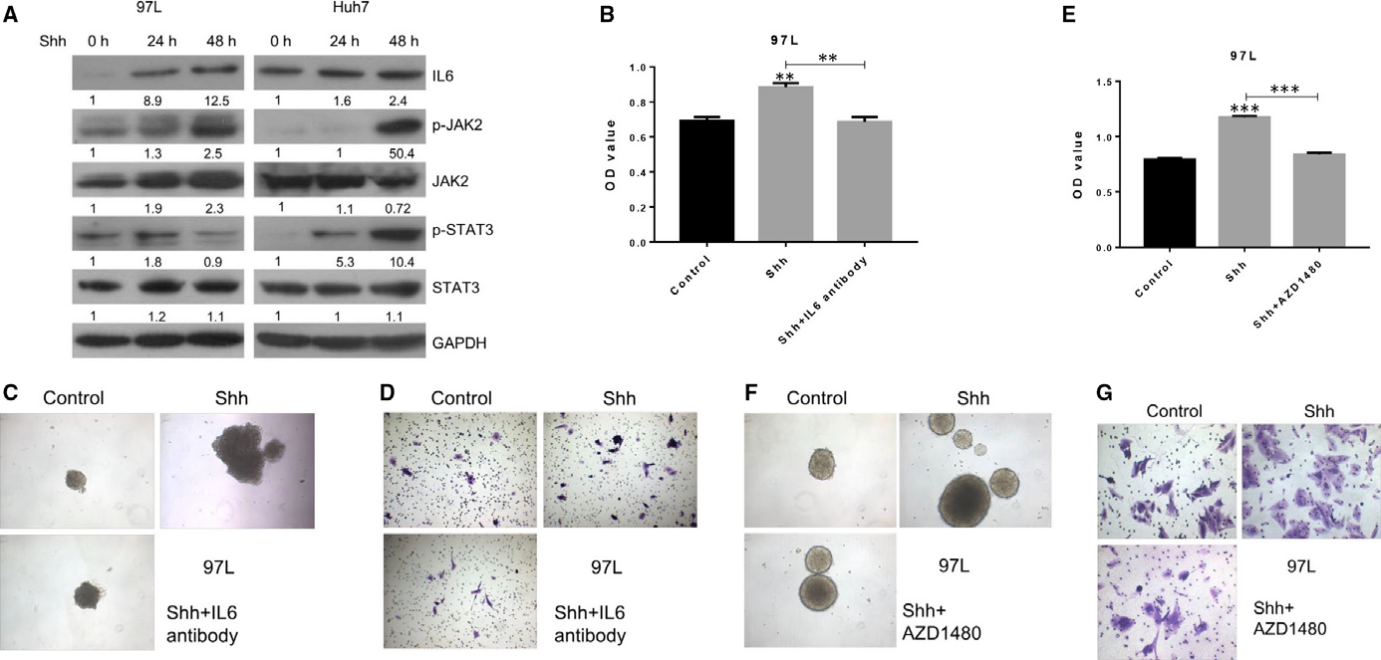
Implications of IL6/JAK2 signalling in CD90+ liver cancer cells. A, Alteration of IL6/JAK2 signalling components in CD90+ 97L and Huh7 liver cancer cells treated with SHH. GAPDH was used as the internal standard. B, Proliferation rates of CD90+ 97L liver cancer cells treated with a combination of SHH and AZD1480 using the MTS assay. C, Sphere formation capacity of CD90+ liver cancer cells treated with SHH and AZD1480. D, Migration capacities of CD90+ liver cancer cells treated with SHH and AZD1480 by the transwell assay. E, Proliferation rates of CD90+97L liver cancer cells treated with a combination of SHH and IL6 neutralizing antibody using the MTS assay. F, Sphere formation capacity of CD90+ liver cancer cells treated with SHH and IL6 neutralizing antibody. G, Migration capacities of CD90+ liver cancer cells treated with SHH and IL6 neutralizing antibody by the transwell assay; SHH: Sonic Hedgehog; IL6: interleukin‐6; p‐JAK2: phosphorylated Janus kinase 2; p‐STAT3: phosphorylated signal transducer and activator of transcription 3; SOX2: Sex Determining Region Y‐Box 2; GAPDH: glyceraldehyde‐3‐phosphate dehydrogenase

For more information about the downstream pathways associated with CD90‐mediated stem cell functioning in liver cancer cells, the IL6/JAK2 signalling components and STAT3 phosphorylation were further analysed in liver cancer cells 97L and Huh7 (Figure 6 and Figure S3). The IL6, phosphorylated JAK2, phosphorylated STAT3 and SOX2 protein levels in 97L cancer cells were greatly elevated by SHH treatment (Figure 6A). Moreover, the proliferation rates of liver cancer cells were also greatly suppressed by treatment with the JAK2 inhibitor and IL6 neutralizing antibody (Figure 6B,E). The sphere formation (Figure 6C,F) and migration capacities (Figure 6D,G) of liver cancer stem cells showed a similar decrease in response to the treatment with IL6 neutralizing antibody and AZD1480.

**Figure 7:**
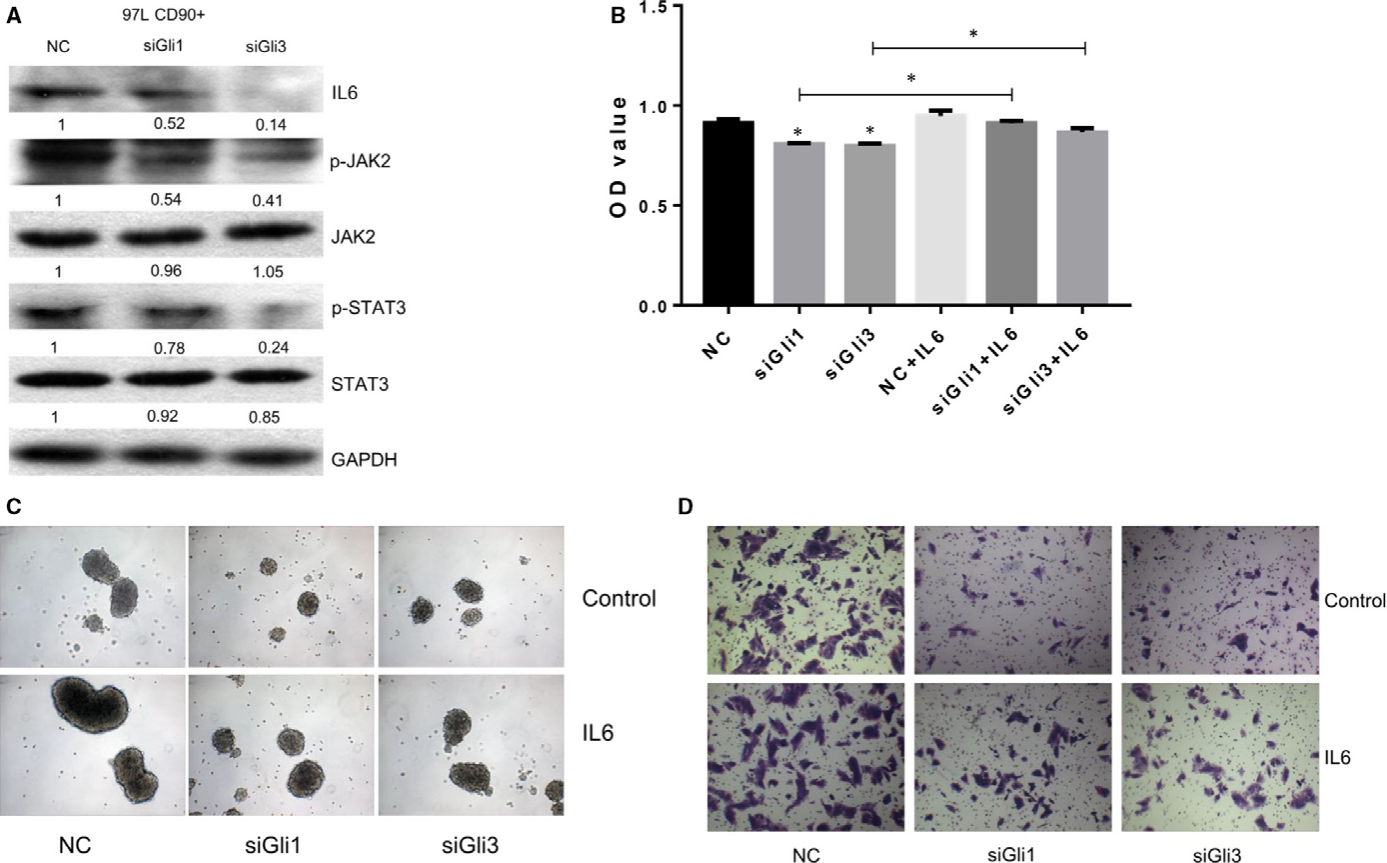
IL6 treatment of CD90+ liver cancer cells. A, IL6/JAK2 signalling components in CD90+ 97L liver cancer cells transfected with Gli and Gli3 siRNAs by Western blotting. B, Proliferation rates of CD90+ 97L liver cancer cells treated with IL6 after Gli1 or Gli3 knockdown using the MTS assay. C, Sphere formation capacity of CD90+ liver cancer cells treated with IL6 after Gli1 or Gli3 knockdown. D, Migration capacities of CD90+ liver cancer cells treated with IL6 after Gli1 or Gli3 knockdown by the transwell assay; IL6: interleukin‐6; p‐JAK2: phosphorylated Janus kinase 2; p‐STAT3: phosphorylated signal transducer and activator of transcription 3; GAPDH: glyceraldehyde‐3‐phosphate dehydrogenase

In contrast, knockdown of Gli1 and Gli3 significantly down‐regulated IL‐6, phosphorylated JAK2, phosphorylated STAT3protein abundances in CD90+ 97L liver cancer cells (Figure 7A). Furthermore, knockdown of Gli1 and Gli3 expression in CD90+ 97L liver cancer cells followed by IL6 treatment can partly reversed the inhibition of cell proliferation, sphere formation capacity and migration capacity caused by Gli1 and Gli3 knockdown (Figure 7B‐D).

Taken together, these results showed that the proliferation, sphere formation and migration capacities of CD90+ liver cancer cells involved activation of IL6/JAK2/STAT3 signalling through the SHH/Gli pathway.

## 4 DISCUSSION

As the characterization of stem cells in various solid tumours, no effort has been spared by the research community to investigate reliable and specific cancer stem cell markers.^4^, ^21^ In liver cancer, the identification of CD133+ cells in hepatocarcinoma cell lines has provided a good start. CD133, a key antigen expressed in the embryonic epithelia of various human organs like the gut, kidney and neural tube, and previously identified as a marker for hematopoietic stem cells, was characterized as a liver cancer cell marker based on the cancer stem/progenitor cell‐like properties of CD133+ cells screened from several liver cancer cell lines, including Huh‐7, SMMC‐7721, HepG2 and PLC 8024.^22^, ^23^, ^24^ However, it has been suggested that CD133 is not a sufficiently sensitive or specific marker of liver cancer stem cells because of the capacity of CD133‐liver cancer cells to generate tumours in immunodeficient mice, among other reasons.^6^, ^23^ To obtain a better definition of the liver cancer stem cell population, CD44, CD24, EpCAM, CD90, CD13, DLK and ALDH1, among others, were identified as potential biomarkers of liver cancer stem cells.^1^ Among this panel of liver cancer stem cell biomarker candidates, CD90 has been considered one of most promising markers because of the highly positive correlation of CD90 expression with the tumorigenicity and metastatic potentials of liver cancer cell lines and their strong capacity to generate liver tumor nodules in mice, which was not observed in CD90^−^ liver cancer cells.^6^ Therefore, in the present study, CD90+ liver cancer stem cells were utilized to better understand the role of stem cells during liver cancer pathological processes.

Although CD90 has already been used as novel target for the development of liver cancer therapy modules,^25^ the molecular mechanisms underlying the association of CD90 with liver cancer stem cell properties remain poorly understood. Inspired by previous reports showing the involvement of SHH signalling in cell proliferation regulation, tumorigenesis and especially liver cancer pathogenesis,^14^, ^15^ we studied the expression of major components of the Shh/Gli signalling pathway in multiple liver cancer cell lines and clinical tissues. The high correlation between CD90 expression and Gli1 and Gli3 expression in the cell lines and patient tissues suggested that SHH/Gli signalling might regulate the stem cell properties of CD90+ liver cancer cells, which was further supported by the analysis of large‐scale liver cancer expression profiles from the TCGA database. To test this hypothesis in liver cancer cell lines, siRNA‐mediated Gli1 and Gli3 knockdown, as well as activation of the signalling pathway using SHH treatment and the combination treatment of CD90 knockdown with SHH treatment, were carried out in the present study. Significant alterations of cell proliferation and migration rates, sphere formation and tumorigenicity capacity induced by knockdown or SHH treatment confirmed the function of SHH/Gli signalling in liver cancer stem cell maintenance mediated by CD90. The expression of SOX2 and NANOG, 2 key stem cell biomarkers regulating cell self‐renewal,^26^, ^27^ further verified the role of SHH/Gli signalling in CD90+ liver cancer stem cell regulation in this study. These results provided novel insights into the molecular mechanisms of CD90‐mediated stem cell property maintenance during liver cancer initiation, progression and also resistance to chemo‐ or radio‐therapy.

In addition, the IL6/JAK2/STAT3 signalling pathway was demonstrated to be involved in CD90+ liver cancer stem cell function modulation in our assays. Previous lines of evidence have shown that activation of the IL‐6/JAK2/STAT3 pathway is closely associated with epithelial–mesenchymal transition and stem cell‐like characteristics, which finally contributes to poor outcomes in patients with various human cancer types.^28^, ^29^, ^30^, ^31^ In liver cancer, application of the JAK2 signal inhibitor has been shown to enhance adriamycin‐induced liver cancer cell ageing, demonstrating the association of the IL6/JAK2/STAT3 signalling pathway with liver cancer pathology.^31^ More importantly, the IL6/JAK2/STAT3 signalling pathway is regulated by GLI1, which maintains STAT3 activation by binding to and activating the IL‐6 gene promoter.^32^ Our discovery of the regulation of IL6/JAK2/STAT3 signalling by the SHH/GLI axis in CD90+ liver cancer cells further broadened our views concerning how CD90 functions in stem cells responsible for liver cancer progression. Furthermore, the IL6/JAK2/STAT3 pathways was also found to be associated with cancer stem cell functions mediated by other stem cell biomarkers, such as CD44,^28^ CD24 33 and EpCAM.^34^ The prevalent functions of IL6/JAK2/STAT3 pathways in multiple stem cell marker‐related processes suggested that this pathway might act as an interacting hub for integrating various stem cell signalling components during cancer stem cell growth and renewal, which merits further investigation.

## 5 CONCLUSION

In summary, we reported herein the highly correlated expression between the cell surface biomarker CD90 and SHH‐related signalling components Gli1 and Gli3 in liver cancer cells and tissues, as well as the mediating roles of SHH/Gli signalling in the regulation of CD90+ liver cancer stem cell function. We also further verified that IL6/JAK2/STAT3 signalling functions downstream of the SHH/Gli pathway in liver cancer stem cells. These findings provided new information about the molecular signalling networks underlying CD90‐mediated stem cell maintenance during liver cancer progression.

## CONFLICTS OF INTEREST

The authors declare that they have no conflicts of interest.

## AUTHORS’ CONTRIBUTIONS

Ketao Zhang, Siyao Che, weidong Wang and Jianping Liu conceived and designed the study, and critically revised the manuscript. Zheng Su, Chuzhi Pan and Shangyou Zheng performed the experiments, analysed the data and drafted the manuscript. Huayao Zhang, Shanglin Yang and Wenda Li participated in study design, study implementation and manuscript revision. All authors read and approved the final manuscript.
