# Supplementary figures and images for: The SHH/Gli axis regulates CD90‐mediated liver cancer stem cell function by activating the IL6/JAK2 pathway
Source: J Cell Mol Med. 2018 May 2;22(7):3679–90. doi: 10.1111/jcmm.13651 (PMC6010714; doi:10.1111/jcmm.13651)

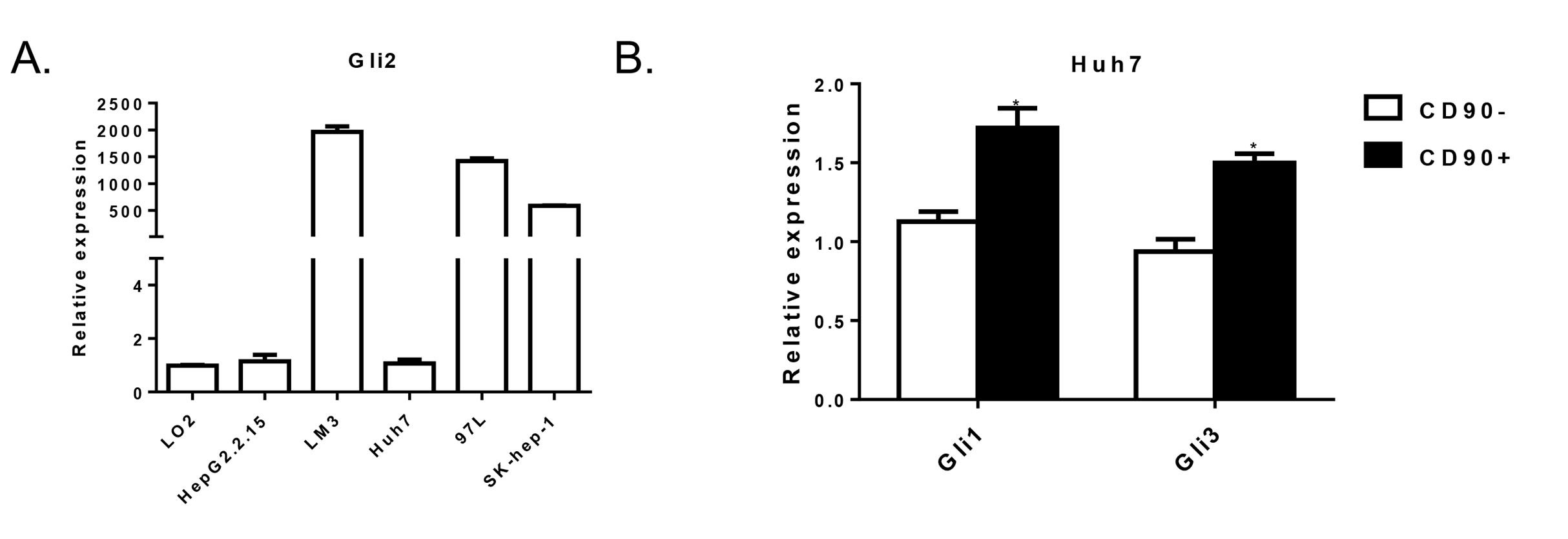

Supplement: Supplementary file 1 [file JCMM-22-3679-s001.tif]

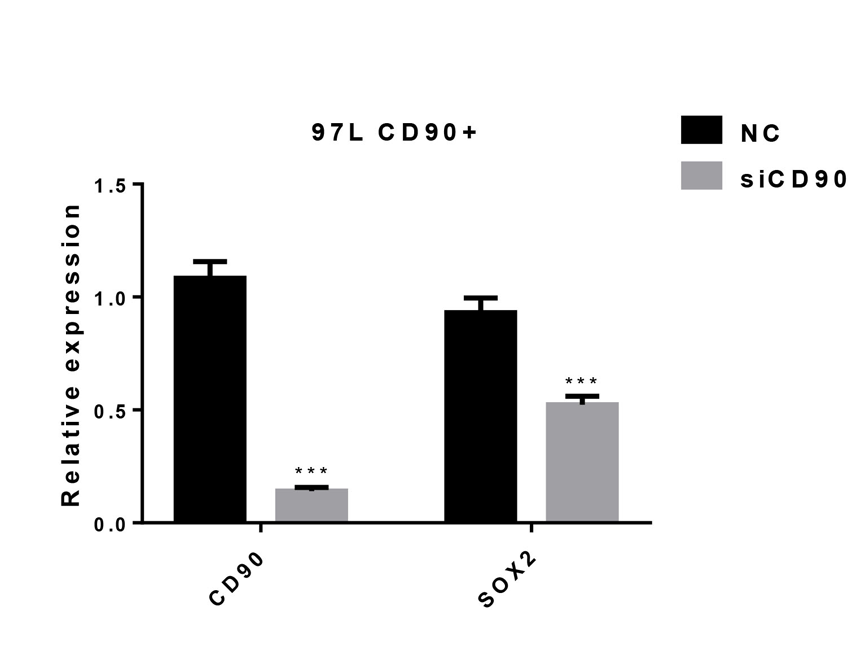

Supplement: Supplementary file 2 [file JCMM-22-3679-s002.tif]

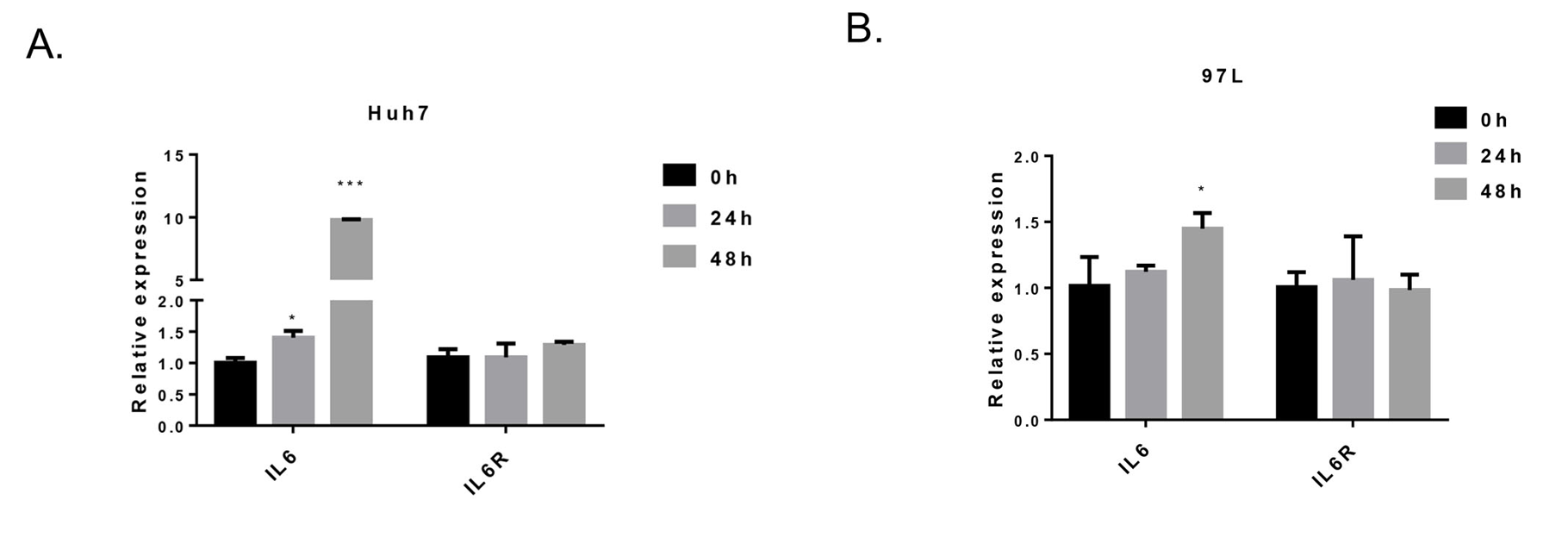

Supplement: Supplementary file 3 [file JCMM-22-3679-s003.tif]
