# Supplementary material for: The SHH/Gli axis regulates CD90‐mediated liver cancer stem cell function by activating the IL6/JAK2 pathway
Source: J Cell Mol Med. 2018 May 2;22(7):3679–90. doi: 10.1111/jcmm.13651 (PMC6010714; doi:10.1111/jcmm.13651)
Supplement: Supplementary file 4 [file JCMM-22-3679-s004.docx]

**Supplemental Figures**

**Supplementary Figure 1: SHH and Gli expression in different liver cancer cells.**

(A) Expression of Gli2 in different liver cancer stem cell lines by quantitative RT-PCR. (C) Expression of Gli1 and Gli3 in CD90-positive and -negative Huh cells by quantitative RT-PCR. SHH: Sonic Hedgehog; Gli1/2/3: Glioma-associated oncogene 1/2/3; * indicates significant differences with p < 0.05.

**Supplementary Figure 2:CD90 knockdown efficiency in CD90+97L liver cancer cells and the SOX expression level.**

**Supplementary Figure 3: Gli1/3, IL6, and IL6R expression in multiple liver cancer cells treated with SHH.**

(B) Expression of IL6 and IL6R in Huh7 cells treated with SHH by quantitative RT-PCR. SHH: Sonic Hedgehog; (C) Expression of IL6 and IL6R in 97L cells treated with SHH by quantitative RT-PCR. Gli1/3: Glioma-associated oncogene 1/3; IL6: interleukin-6; IL6R: interleukin 6 receptor; * indicates significant differences with p < 0.05.
